# UC2‐ESP: A general‐purpose framework for open‐source microscopy control

**DOI:** 10.1111/jmi.70147

**Published:** 2026-07-21

**Authors:** Benedict Diederich, Ingo Fuchs, Haoran Wang, Holger Bierhoff, Christian Kuttke, Rainer Heintzmann

**Affiliations:** ^1^ Leibniz Institute of Photonic Technology Jena Germany; ^2^ openUC2 GmbH Jena Germany; ^3^ Institute of Biochemistry and Biophysics, Center for Molecular Biomedicine (CMB) Friedrich Schiller University Jena Germany; ^4^ Leibniz Institute on Aging Fritz Lipmann Institute Jena Germany; ^5^ Institute of Physical Chemistry and Abbe Center of Photonics Friedrich Schiller University Jena Jena Germany

**Keywords:** ESP32, microscopy control software, open hardware, Python, smart microscopy

## Abstract

Building the optical setup for investigating biological questions comes with challenges. A major such challenge is setting up and synchronizing the control of multiple hardware components such as stages, cameras and lasers. With UC2‐ESP, we present a low‐cost electronics system powered by the ESP32 microcontroller (∼$10), designed as an accessible, open‐hardware alternative to commercial controllers for custom and open‐source microscopy setups. Our system can interface with stepper motors, direct current motors, lasers (transistor‐transistor logics, TTL or pulse width modulation, PWM), light‐emitting diodes and analog voltage outputs (galvo mirrors and LED current control), allowing precise control over microscopy hardware. The platform is highly flexible, supporting custom pin configurations and multiple communication interfaces such as Bluetooth, universal serial bus (USB‐serial) and HTTP via a built‐in web server. A PlayStation controller can be used for haptic hardware manipulation, whereas commands are transmitted in a human‐readable JSON format to ensure modularity and extensibility. The firmware is designed to receive parameters and execute actions dynamically, supporting complex control loops such as motor homing, stage scanning and temperature regulation via integrated controllers. Furthermore, the system integrates seamlessly with ImSwitch as well as Micro‐Manager and offers a browser‐based control tool using Web Serial. This open‐source firmware enables microscopy research groups to develop custom setups and expand functionality efficiently, at low cost and high flexibility.

## 1 THE NEED FOR CONTROL—IN MICROSCOPY

Modern microscopes have remained conceptually similar for more than a century, aiming to provide insights at the microscopic scale for biology, life sciences and materials science. Yet present‐day instruments are no longer just optical devices: they increasingly resemble compact, application‐specific robots. Advanced systems contain motorized *XY* and *Z* stages, piezo drives, galvanometric mirrors, adaptive optics, heaters, cameras and multiple illumination sources ranging from light‐emitting diodes (LEDs) to pulsed lasers. All these subsystems must interact at millisecond precision, much like the coordinated motion control in 3D printers. The ability to perform accurate time‐lapse experiments, high‐speed imaging or super‐resolution methods requires tight synchronization between illumination, detection and mechanical movement.^1^, ^2^, ^3^, ^4^, ^5^

This complexity places microscope developers in a role akin to ‘full‐stack developers’: They must combine optical design, mechanical engineering, electronics integration, low‐level firmware development and high‐level software for experimental workflows.^6^ The final hurdle is often the orchestrated digital control of all components, for example, moving a stage while simultaneously triggering a laser and synchronising camera exposure to minimize photobleaching. Commercial controllers and DAQ cards exist, but they are expensive, often closed source and rarely integrate well with modern workflows that rely on Python‐based analysis, machine learning or real‐time feedback.

The maker community has shown that mass‐produced, off‐the‐shelf hardware can be repurposed: Budget 3D printers provide precise motorized rails, and by replacing the extruder with a camera or attaching entirely new tool heads, platforms like the EnderScope,^7^ HistoEnder^8^ or the Opentrons‐contained microscope^9^ demonstrate how one can build task‐specific scientific instruments at a fraction of the cost of bespoke instruments. These printers rely on established G‐code standards and mature motion‐planning firmware, such as GRBL, Marlin or Klipper. However, while highly optimised for extrusion and motion control, these large codebases are difficult to adapt for microscope‐specific components like multiple light sources, filter wheels or sensitive detectors. Unlike 3D printing, microscopy lacks a universal control language: Every stage, laser, camera or piezo typically comes with its own proprietary driver or dynamic linked library, often locked behind closed USB protocols. Therefore, scientists who build custom microscopes must be familiar with a variety of device‐specific libraries and programming patterns in order to ultimately obtain microscopic images or create more complex workflows. Custom prototypes complicate matters further: One setup may only require a TTL pulse to gate a diode laser, while another light‐sheet system may need four high‐current stepper channels for sample scanning plus an additional axis for automated focusing. Off‐the‐shelf controllers rarely expose ‘hackable’ interfaces, and discontinued vendor hardware often ends up as electronic waste.

These issues, combined with the necessity to digitise our own modular UC2 microscopes, motivated the design of UC2‐ESP: a modular, hardware agnostic, fully open‐source firmware and electronics framework. Our approach is guided by the following design goals:

1. Cross‐platform operation regardless of host operating system (compatible with Windows, macOS and Linux; can also be powered via USB or battery packs for portable field setups),
2. Stand‐alone capable hardware control,
3. Broad support for off‐the‐shelf components common in microscopy hardware,
4. Deployment on inexpensive, readily available microcontrollers,
5. A browser‐based WebSerial tool for interactive testing and debugging, requiring no dedicated desktop application installation,
6. Pre‐built firmware variants and a web‐based flashing workflow for supported boards, reducing initial setup effort, and
7. A documented JSON/REST API designed to integrate with diverse experimental workflows and control software.

By combining configurable hardware modules (stepper drivers, PWM laser controllers, temperature regulators) with the ESP32 microcontroller, UC2‐ESP^10^ consolidates drivers for lasers, stages, heaters, sensors and auxiliary opto‐electronics into a single lightweight binary. Devices are exposed via Wi‐Fi, Bluetooth, controller area network (CAN), I^2^C or USB under a unified JSON/REST command layer. Instead of needing one controller or port per device, the system reduces the hardware footprint to a single interface while remaining extensible and hackable. This allows both novice and expert users to rapidly build, modify or repurpose experimental setups and integrate them seamlessly with environments such as Python or GUI platforms like ImSwitch.^11^

### 1.1 Scope, trade‐offs and non‐goals

1. Timing determinism is millisecond‐class, not sub‐microsecond as on FPGA/DAQ.
2. Analog bandwidth is limited to the ESP32 on‐board digital–analog converter (DAC) (or external SPI‐associated hardware and their bus speed).
3. Communication‐wise, hard real‐time guarantees are not provided over Wi‐Fi/BT/USB‐Serial.
4. It is not intended for high‐throughput screening or kHz‐rate triggering applications.
5. It complements rather than replaces NI DAQ, FPGA, and vendor controllers.

## 2 METHODS

### 2.1 System architecture

**FIGURE 1:**
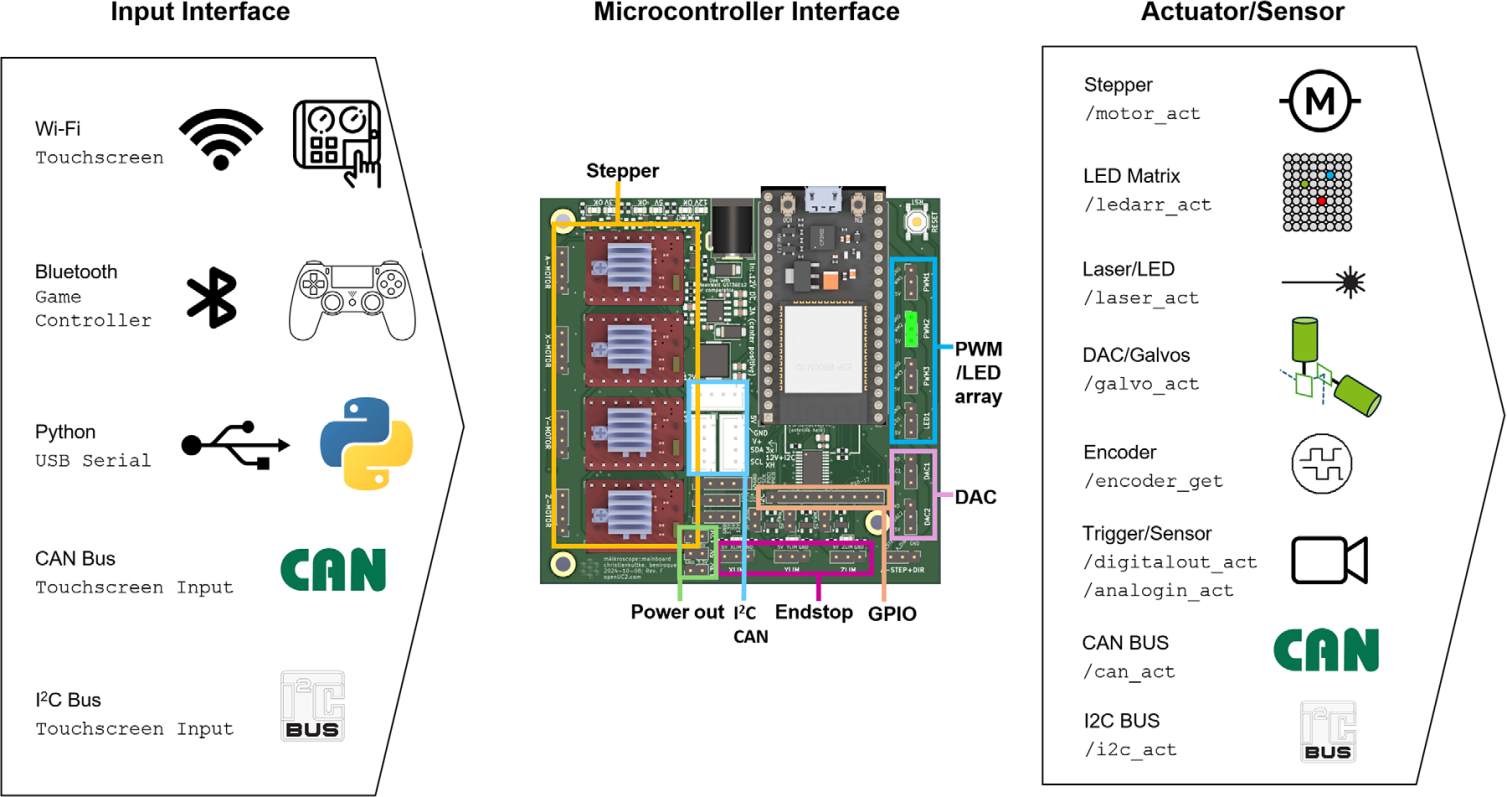
Input–output diagram. The microcontroller accepts various input interface (left) and can drive different actuators and sensors (right).

We chose the ESP32 as the central element for driving various electronic components—such as stepper motors, LED arrays (e.g., for phase‐contrast microscopy), TTL/PWM signals for laser control, or sensor evaluation via I^2^C and distributed operation via CAN bus. This compact microcontroller comes with a wide range of interfaces (UART, Wi‐Fi, Bluetooth, I^2^C, CAN bus, etc.) and benefits from a large open‐source development community in which many code examples and drivers are available.^12^ As the ESP32 lacks the necessary power electronics to directly drive high‐current components such as stepper motors or lasers, additional hardware is required. We, therefore, provide two complementary solutions. The first leverages standardized adapter boards widely used in CNC systems and 3D printers—most notably the CNC Shield v3 (Protoneer) combined with an ESP32‐WEMOS D1 (various manufacturers). To accommodate different hardware configurations, pin assignments are decoupled from the main firmware, allowing flexible customization. The second solution is a series of custom‐developed extension boards (i.e., UC2e), designed specifically for microscopy setups. This board integrates support for different communication protocols and is optimized for the combination of actuators, light sources and sensors typically required in modular (UC2) microscopes as shown in Figure 1.

**FIGURE 2:**
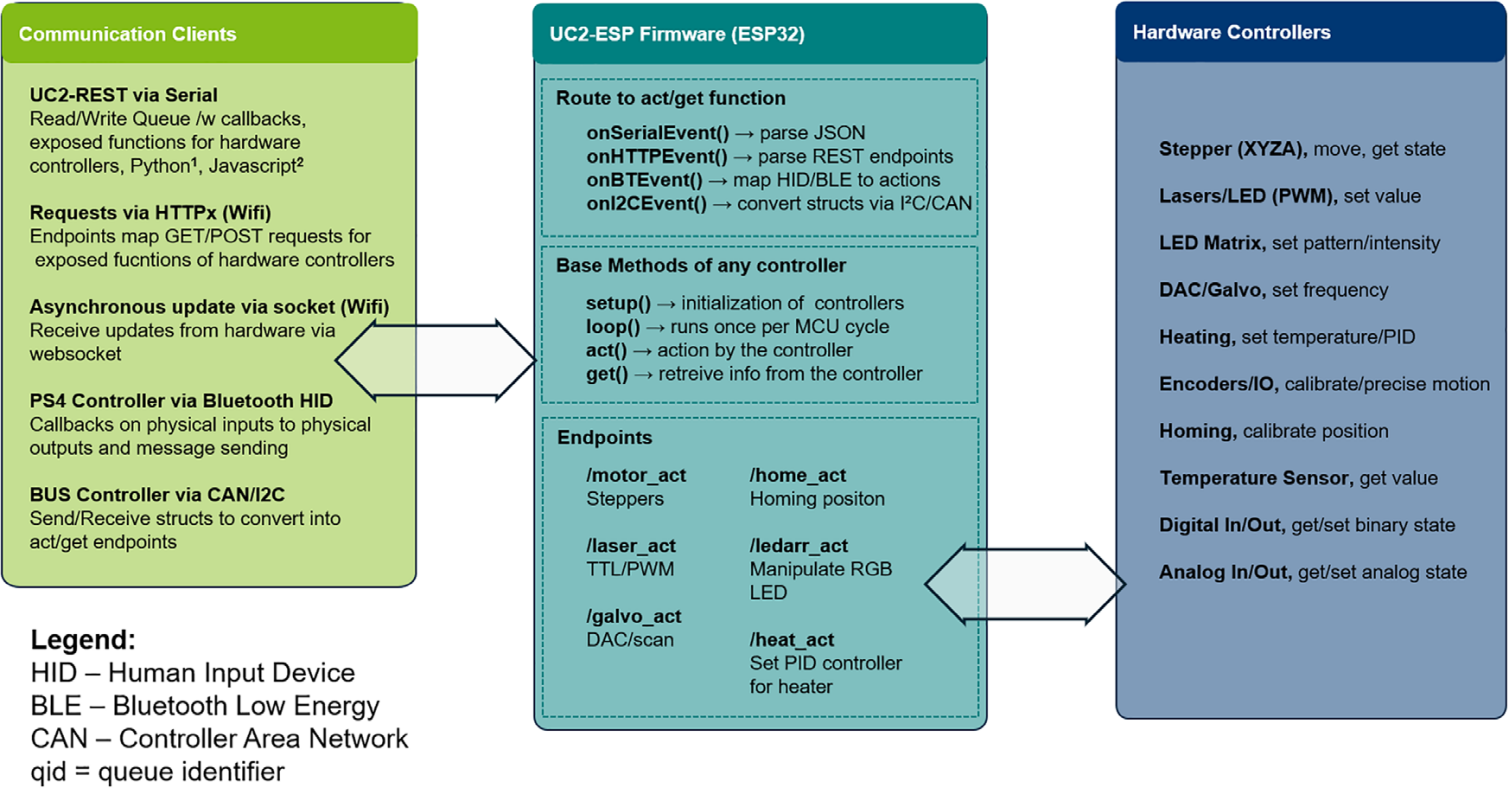
Firmware structure. The firmware supports multiple communication clients and decodes task functions into executable actions for the hardware.

UC2‐ESP distinguishes time‐critical hardware synchronization, which is handled on the microcontroller itself, from user‐directed parameter updates that can tolerate network latency. The latter are delivered asynchronously over Wi‐Fi, USB‐Serial or Bluetooth. For example, a PlayStation 4 joystick can adjust laser power in real time via Bluetooth, a smartphone can send the same command through the ESP32's access‐point mode, and a Python script can stream updates over a wired serial connection. All messages use a self‐descriptive JSON syntax and a REST‐like endpoint scheme (e.g., */motor_act*), making the API both human‐readable and straightforward to extend. Although JSON adds a modest payload overhead, it simplifies debugging and reduces the integration burden on client software (Figure 2).

The firmware is built using PlatformIO,^13^ an open‐source community‐based framework to write and compile firmware for different microcontrollers,^13^ providing a reproducible development environment and simplifying the integration of both the ESP‐IDF toolchain^12^ and Arduino components.^14^ The programming consists of several modules, a structure that follows the classic Arduino approach with a *setup()* function (initialization) and a *loop()* function for each such module. The globally running programming loop runs through individual module loops consecutively. This allows multiple tasks to run concurrently—for example, when using a closed‐loop control mechanism for stepper motors. Each module's setup routine is executed at initialization.

To keep the memory footprint small and tailor the configuration to various applications, modules and communication interfaces are switched on or off using preprocessor directives (#define). Wi‐Fi, Bluetooth and USB‐serial can, thus, run simultaneously or be used exclusively to optimize performance. Certain tasks can also be offloaded to satellite boards connected via I^2^C or CAN bus—for instance, handling complex stepper motor control loops, including acceleration profiles and time‐critical closed‐loop regulation. In such cases, the master board (ESP32) merely handles the communication, whereas the specialized secondary board carries out the control tasks. This modular principle conserves memory and computing resources on the main system while still enabling efficient implementation of sophisticated control requirements.

**FIGURE 3:**
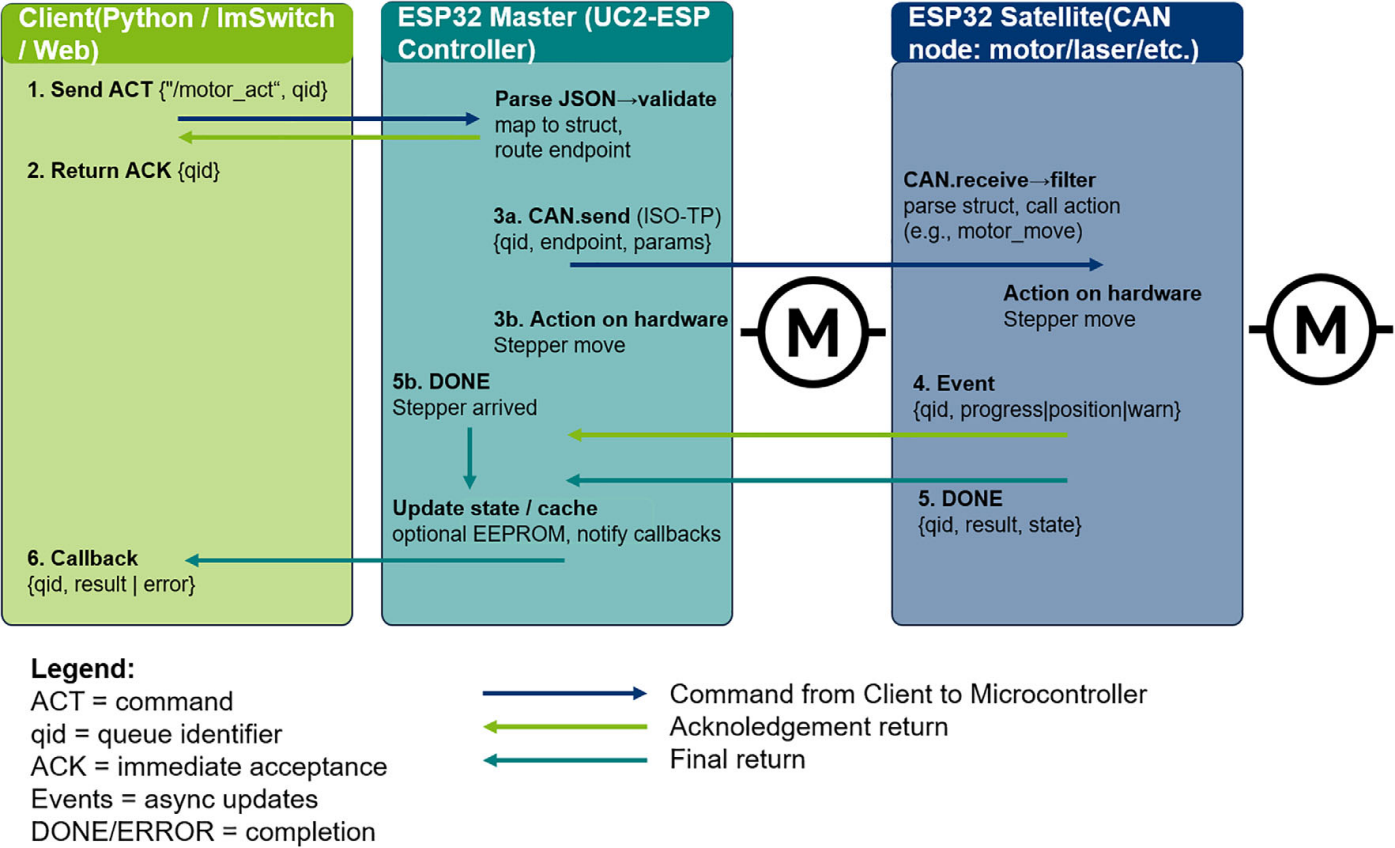
Task flow of the firmware. An operation initiated by the client generates an ‘ACT’ message, which is transmitted to the firmware to request execution of a function. The firmware parses the received JSON string, extracts the task and translates it into a hardware‐specific action. This command is, then, delivered to the corresponding hardware module for execution. Upon successful completion, the firmware transmits a response message back to the client, thereby confirming task execution.

Each request sent from the host to the ESP32 firmware includes a unique request ID (qid) also visualized in Figure 3. Upon receiving a well‐formed JSON request, the ESP32 immediately returns an interim response echoing the qid, asynchronously before executing the command. This acknowledgement ensures that malformed or lost requests can be identified; for example, in case a request cannot be parsed (e.g., invalid JSON), the ESP32 responds with a negative qid to indicate an error.

Once the request has been fully executed, the ESP32 issues a final response associated with the same qid. For example, if the host requests a motor to move to a new position, the interim acknowledgement confirms receipt of the request, and the final response confirms that the motor has reached its target (or that the request was superseded by a newer one).

In addition to responding to host‐initiated requests, the ESP32 can also send asynchronous updates to the host, such as reporting a new motor position when moved by an external input (e.g., joystick). On the host side, these unsolicited messages can be bound to callback functions, allowing the system state to remain synchronized without constant polling. In effect, the communication model combines a traditional request/response pattern with event‐driven updates, giving both sides the ability to exchange messages as needed.

## 3 FIRMWARE FEATURES

### 3.1 Rest‐like interface

The firmware's API is inspired by common REST practices, offering a unified, platform‐independent interface for controlling actuators and reading sensors. Two types of commands exist: an ‘ACT’ (analogous to a POST) that triggers a function and a ‘GET’ (similar to a GET request) that retrieves current system states. An example ‘ACT’ command to move two stepper motors simultaneously by a given number of steps might look like the following:




{
“task”: “/motor_act”,
“motor”: {
“steppers”: [
{“stepperid”: 1, “position”: 10000, “speed”: 5000, “isabs”: 0, “isaccel”: 0},
{“stepperid”: 3, “position”: 10000, “speed”: 5000, “isabs”: 1, “isaccel”: 0}
]
}
}



In this case, motor 1 moves 10,000 steps at 5000 steps/s in a non‐accelerated, relative motion, whereas motor 3 moves to position 10,000 with the same parameters. The firmware parses the JSON string, starts the motors in a non‐blocking manner and immediately returns a success message. A second message is sent once the final positions are reached, including updated positions. An optional queue ID (qid) can be included to correlate commands and responses.

A ‘GET’ request, such as




{“task”: “/motor_get”, “qid”:1}

retrieves the current state of each motor (e.g., enabled status and position):




{
“motor”:
{“steppers”:[
{“stepperid”:0,“position”:0,“trigOff”:0,“trigPer”:‐1,“trigPin”:‐1,
“isStop”:0,“isRunning”:0,“isDualAxisZ”:0,“isforever”:0,“isen”:0,“stopped”:1},
{“stepperid”:1,“position”:188990,“trigOff”:0,“trigPer”:‐1,“trigPin”:‐1,“isStop”:0,“isRunning”:0,“isDualAxisZ”:0,“isforever”:1,“isen”:0,“stopped”:1},
{“stepperid”:2,“position”:117315,“trigOff”:0,“trigPer”:‐1,“trigPin”:‐1,“isStop”:0,“isRunning”:0,“isDualAxisZ”:0,“isforever”:1,“isen”:0,“stopped”:1},
{“stepperid”:3,“position”:‐70926,“trigOff”:0,“trigPer”:‐1,“trigPin”:‐1,“isStop”:0,“isRunning”:0,“isDualAxisZ”:0,“isforever”:1,“isen”:0,“stopped”:1}]},“qid”:1
}



Similar requests can quickly poll sensor data, making it straightforward to integrate real‐time monitoring of temperature, light intensity or other parameters. This way, host software can work asynchronously by relying on events sent by the microcontroller or synchronously, by actively polling the microcontroller's state (Supporting Information S1).

### 3.2 Connecting multiple controllers on the microcontroller

Within the firmware, different modules can be linked to enable automated workflows. For instance, temperature control,^15^ where the reading from a digital temperature sensor (e.g., DS28xx) is fed into a PID controller to regulate a PWM heater, maintaining a stable environment (e.g., for a stage‐top incubator). Similarly, a home‐position routine connects a digital input controller (endstop) to a stepper motor controller, instructing the stepper to move until the endstop is triggered and then reset its position to zero. The resulting controller provides a new REST‐like endpoint (inspired by lab‐things^16^), whereas the execution and computation of control actions (e.g., in the case of the temperature control) are fully carried out on the microcontroller, hence saving resources when sending/receiving control commands. An exemplary homing command might look like the following:




{
“task”: “/home_act”,
“home”: {
“steppers”: [
{“stepperid”: 2, “timeout”: 20000, “speed”: 15000, “direction”: ‐1, “endposrelease”: 3000}
]
}
}



This instructs the firmware to move stepper 2 at 15,000 steps/s in the specified direction, check for the endstop and then back off by 3000 steps once triggered, demonstrating how real‐time control routines are directly executable on the microcontroller unit (MCU) level through the same REST‐like interface.

### 3.3 Integration of satellites via I^2^C

To support more complex workflows and reduce the computational overload on the main controller, UC2‐ESP firmware allows external devices to be networked over I^2^C. As I^2^C is a standard interface for sensors and actuators, integrating new peripherals such as a temperature or pressure sensor becomes straightforward. Developers can simply attach the device on the I^2^C bus and access it via the existing JSON/serial interface without major firmware changes.

Beyond simple peripherals, UC2‐ESP also supports the creation of custom I^2^C ‘satellites’. These can be secondary microcontrollers such as another ESP32 configured as I^2^C slaves with their own device address. A typical use case is offloading real‐time, closed‐loop tasks such as detailed stepper acceleration profiles to a satellite board, whereas the primary ESP32 remains responsible for high‐level coordination and communication (Wi‐Fi, Bluetooth, USB). This division of labour reduces both memory and processing load on the primary MCU while still exposing the satellite's functionality (e.g., motor control) through the same unified JSON/I^2^C interface. In this way, UC2‐ESP makes it equally simple to integrate commodity I^2^C devices or to extend the system with custom controllers that interoperate seamlessly with the rest of the firmware.

### 3.4 Integration of satellites via CAN

Unlike I^2^C, where the master must actively poll each slave for status updates, a CAN supports true bidirectional messaging: any node can transmit, and all nodes on the bus receive the frame but only act on messages addressed to them. Widely adopted in the automotive industry, CAN uses a differential signal pair that is tolerant of electrical noise and higher supply voltages, ideal for setups where 12 V motor drivers share cabling with low‐level logic. The ESP32 includes a native CAN (TWAI) peripheral, and a transceiver module is used to translate between logic and bus levels.

In UC2‐ESP we assign CAN identifiers (IDs) to each class of component (e.g., master node: 0×00, motors: 0×10–0×19, lasers: 0×20–0×29). Every node implements both transmit and receive handlers. Upon receiving a message, each device filters the frame headers and processes only messages with IDs relevant to its function, thereby avoiding unnecessary parsing of unrelated traffic. The design follows a request/response pattern for most interactions: the ‘master’ (e.g., a controller interfacing with Python or ImSwitch) sends requests to a satellite (e.g., move motor *X* to position *N*), and the addressed device responds when the request is acknowledged or completed. However, satellites can also issue messages on their own, for instance, a motor controller broadcasting that it has reached its target position or a sensor publishing a new measurement without being explicitly polled. This mix of directed requests and asynchronous state updates reduces bus traffic while keeping all participants synchronized.

Because the JSON‐based UC2‐ESP protocol often exceeds the 8‐byte payload of a single CAN frame, we employ ISO‐TP^17^ segmentation to split large commands across multiple frames, which the receiver then reassembles into a single action. Sending and receiving tasks run concurrently in separate queues, enabling reliable handling of simultaneous traffic. Broadcasts (messages intended for all nodes) are supported through a reserved ID. Figure 3 illustrates a mixed UC2‐ESP network where motors, sensors, and lasers share the same two‐wire bus, coordinated through ID assignment.

### 3.5 Integration with software

From the outset, the firmware has been designed to be as user‐friendly and straightforward as possible, minimizing additional software requirements. Because the ESP32 supports various communication methods (HTTP requests, USB serial and Bluetooth), users can interact with the device in multiple ways.

UC2‐ESP exposes a unified JSON/REST firmware API that can be reached through several client layers, each targeting a distinct use case. Micro‐Manager integration is most appropriate when users already operate within an established Micro‐Manager workflow and require GUI‐driven acquisition, device abstraction, and compatibility with existing Micro‐Manager plugins and scripts. ImSwitch integration is better suited for Python‐native environments where researchers need programmable smart‐microscopy loops, machine learning–based feedback or rapid modification of acquisition pipelines. The browser‐based WebSerial tool requires no software installation and is intended for interactive debugging, educational workshops and initial hardware bring‐up; it is not designed for high‐throughput automated acquisition. The ESP32‐hosted HTTP endpoints provide a lightweight control path for IoT‐style deployments, smartphone‐based demonstrations or embedded scenarios where a full desktop environment is unavailable. All four layers share the same firmware API, so hardware can be controlled and tested at any level without firmware changes.

1. **Serial interface**
  The simplest and most robust method involves a wired USB‐Serial connection, allowing direct communication between the host software (e.g., Python, ImSwitch, Micro‐Manager and JavaScript) and the microcontroller. We provide a Python‐based library (UC2‐REST^18^) that wraps each endpoint or device into a dedicated function call, which, then, generates the required JSON string. For example, a function call in Python might look like move_stepper(stepper_id = 1, distance = 1000), which internally constructs the JSON command and sends it to the firmware. This library also supports optional callbacks for events such as target position reached or updated sensor readings.
2. **Browser‐based WebSerial**
  To avoid any installation overhead, we have developed an online control tool that runs in a standard web browser and communicates with an ESP32 master using WebSerial (Youseetoo.github.io). This interface provides a simple GUI for controlling motors, LEDs and lasers without needing to install drivers or additional software. WebSerial allows direct communication with the ESP32's USB‐Serial port, so end users can open a web page, connect to the device and start issuing commands in real time. Through GitHub Actions, the firmware can be compiled automatically and hosted on a static (GitHub) webpage, making new releases instantly accessible. The ESP32 Flashing tool, based on work from the Home Assistant community,^19^ makes use of the newly established Web Serial Standard and helps the user to install firmware on their board variant.
3. **Integration with ImSwitch**
  ImSwitch is a Python‐native microscope control framework designed for flexible, scriptable acquisition pipelines. The UC2‐ESP ImSwitch driver exposes stages, lasers and LEDs as native ImSwitch hardware objects, enabling automated imaging routines such as multi‐position tiled acquisitions, *Z*‐stack collection and closed‐loop feedback experiments where laser intensity or stage position is adjusted in response to live image analysis. This integration is particularly suited to smart‐microscopy workflows and rapid pipeline prototyping, because experimental logic can be modified in Python without recompiling firmware. ImSwitch supports all hardware functionalities that are exposed by the microcontroller serial interface, which also includes the galvo or LED matrix interface.
4. **Integration with Micro‐Manager**
  Similar to the integration with ImSwitch using Python, a dedicated C++ device driver has been developed to interface with Micro‐Manager in a manner akin to a ‘hub device’. Lasers, stages and additional sensors can be controlled via serial commands passed directly between Micro‐Manager and the ESP32. This design makes it easy to integrate UC2‐ESP hardware into existing microscopy workflows without extensive software modifications.
5. **Integration as a web‐request‐based control**
  The ESP32 is well known for its ability to be used in Internet of Things applications (IoT) due to its internal Wi‐Fi module. We implement a simple web server that exposes the different endpoints that control the hardware as http‐request endpoints. The ESP32 can act as an access point or connect to an available Wi‐Fi network. Additionally, a static webpage shipped via this web server renders buttons controlling the hardware. This is very helpful for automated control of a microscope, with the smartphone camera acting as the acquisition device.
6. **Bluetooth controller‐based control**
  In addition to Wi‐Fi, the ESP32 also offers the use of Bluetooth. For this, we implemented a library that can connect to various human interfacing devices, where buttons and joysticks (e.g., from the PS4 controller) are then mapped to hardware functions. This realizes an intuitive control of the microscope when, for example, searching the sample area for a specific feature by steering the joystick.

Overall, these integrations ensure that users can operate the firmware in environments ranging from lightweight browser‐based setups to comprehensive imaging platforms, all while relying on the same unified JSON‐based control structure and the same codebase.

### 3.6 Time‐critical processing of hardware control commands in a sequence

Although UC2‐ESP already permits millisecond‐level coordination of motors, lasers and cameras, it does not yet expose a generic, user‐defined ‘timeline engine’ that would let a client upload arbitrary synchronization table with conditional branches or time‐outs. Time‐critical routines are instead hard‐coded in firmware: For the galvo scanner, for example, a dedicated for loop driven by a hardware timer toggles SPI‐controlled DAC voltages at a fixed pixel dwell time, whereas the *XY* stage scanner moves through a list of coordinates and emits a camera‐trigger pulse at each stop. These blocks run outside the main task, rely on the ESP32's deterministic timer modules, and may employ lightweight ISRs (interrupt service routines) to flip pins precisely without jeopardising the RTOS scheduler. The user can populate such blocks at run time by passing a JSON structure, for example,




{
“task”: “/motor_act”,
“stagescan”: {
“coordinates”: [{“x”:100,“y”:200}, {“x”:300,“y”:400}],
“tPre”:50, “tPost”:50,
“illumination”:[50,75,100], “led”:100
}
}



Or record a linear macro of several commands and replay it multiple times:




{
“tasks”:[
{“task”:“/motor_act”,“motor”:{“steppers”:[{“stepperid”:1,“position”:1000,“speed”:20000,“isaccel”:1,“accel”:500000}]}},
{“task”:“/laser_act”,“LASERid”:3,“LASERval”:200},
{“task”:“/laser_act”,“LASERid”:3,“LASERval”:0},
{“task”:“/state_act”,“delay”:1000}
],
“nTimes”:2
}



During execution these routines are blocking: The serial interface is muted until completion to guarantee timing accuracy. In practice this approach delivers micro‐ to millisecond synchrony for common tasks (galvo line scans, grid acquisitions and stage‐top autofocus), but extending it to fully programmable, conditional timelines remains future work. Planned improvements include a lightweight on‐board scheduler that would accept event tables with absolute or relative timestamps, timeout logic and branching conditions which would enable complex experiment choreography without recompiling firmware while still leveraging hardware timers and ISR hooks for sub‐millisecond determinism. This is more suitable for FPGA‐based approaches in microFPGA.^20^

**FIGURE 4:**
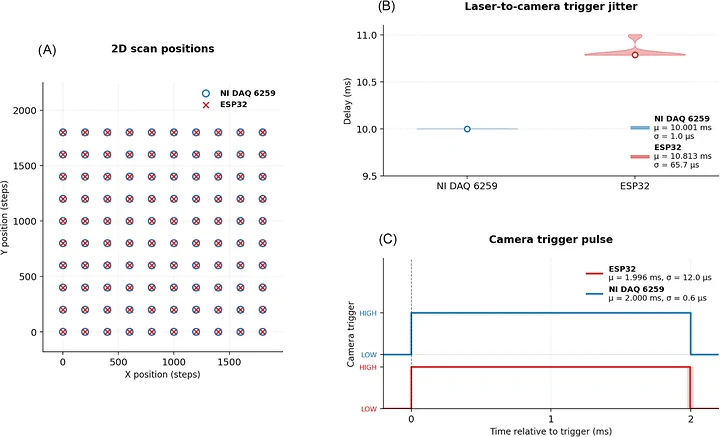
Performance comparison between NI DAQ 6259 and UC2‐ESP during a 10 × 10 raster stage scan. (A) *XY* stage positions at each of the 100 acquisition points, reconstructed from the stepper motor step and direction signals captured by the logic analyser. Coordinates are independently normalized to each system's own origin. Both systems reproduce the same regular grid geometry across the full scan area. (B) Laser‐to‐camera trigger delay distribution for each system, visualized as a kernel density estimate (violin). The hollow circle marks the median value. NI DAQ 6259: *μ* = 10.001 ms, *σ* = 1.0 µs; ESP32: *μ* = 10.813 ms, *σ* = 65.7 µs. (C) Camera trigger pulse for each system shown as a dual‐channel schematic. The shaded band around the falling edge represents ±2*σ* timing jitter on the pulse width. NI DAQ 6259: *μ* = 2.000 ms, *σ* = 0.6 µs; ESP32: *μ* = 1.996 ms, *σ* = 12.0 µs. The NI DAQ 6259 was operated using a pre‐compiled buffered digital output waveform written to the onboard buffer prior to scan execution. The UC2‐ESP was controlled via a single JSON‐based *stagescan* command over USB serial.

To quantitatively characterize the timing and spatial performance of the UC2‐ESP system relative to a professional data acquisition system, a 10 × 10 raster stage scan was performed on both platforms using an NI DAQ 6259 (National Instruments) as the reference. In this scan, the *X*‐axis motor traversed 10 positions per row with 200 steps per grid interval, while the *Y*‐axis advanced by one row after each complete *X*‐axis sweep, following a snake pattern in which the *X*‐axis direction alternated between consecutive rows. At each of the 100 grid positions, the laser was activated via PWM, followed by a pre‐trigger settling delay of 10 ms, after which the camera trigger signal was issued and the laser was switched off after a 50 ms exposure window. All digital signals, including *X* and *Y* motor step and direction signals, laser PWM and camera trigger, were captured simultaneously using a Saleae Logic16 digital analyser (Saleae), and edge timestamps were extracted for subsequent analysis. The resulting timing and spatial metrics are presented in Figure 4.

The *XY* stage trajectories for both systems are shown in Figure 4A, which plots the stage positions at each of the 100 acquisition points as reconstructed from the motor step and direction signals recorded by the logic analyser. Both the NI DAQ 6259 and the UC2‐ESP reproduce the same regular 10 × 10 grid, confirming that the ESP32‐based firmware accurately drives the stage through the intended raster scan geometry.

The NI DAQ 6259 was operated using a pre‐compiled buffered digital output waveform, in which the entire scan sequence was written to the DAQ onboard buffer as a single binary transfer prior to execution, with the hardware clock clocking out every sample at 100 kHz autonomously without real‐time PC intervention. The laser‐to‐camera trigger delay showed a mean of 10.001 ms with a standard deviation of 1.0 µs, and the camera trigger pulse width was 2.000 ms with a standard deviation of 0.6 µs.

The UC2‐ESP system was controlled via a single JSON‐based stagescan command transmitted over USB serial, with the firmware executing the scan on‐device. The laser‐to‐camera trigger delay showed a mean of 10.813 m, representing a systematic offset of 0.813 ms above the nominal 10 ms target, with per‐position variability of 65.7 µs (1*σ*). The camera trigger pulse width measured 1.996 ms with a standard deviation of 12 µs. The laser‐to‐camera trigger delay distributions for both systems are compared in Figure 4B, and the corresponding camera trigger pulse widths with ±2*σ* timing jitter bands are shown in Figure 4C.

The observed timing variations are consistent with the millisecond‐level scheduling resolution described in the firmware architecture and remain well within the requirements of standard fluorescence microscopy workflows where camera exposure times typically range from tens to hundreds of milliseconds. In contrast to the DAQ approach, which requires the entire scan waveform to be recomputed and re‐uploaded for every parameter change, the UC2‐ESP system accepts runtime‐configurable JSON commands, enabling flexible and interactive experimental control without firmware recompilation. Conversely, applications requiring sub‐microsecond timing determinism, such as MINFLUX‐class single‐molecule localization or high‐speed point‐scanning confocal microscopy, remain better served by FPGA‐based controllers.

## 4 USE CASE

The UC2‑ESP firmware has already been deployed in a range of open‑hardware and commercial instruments, highlighting its versatility across very different imaging modalities and actuator‑sensor constellations. Below we name a few use cases where the firmware was used under different platform‐io configurations (noted in the paragraphs).

### 4.1 Structured‑illumination module on a commercial stand (openSIMMO)—DIY and off‐the‐shelf automation

**FIGURE 5:**
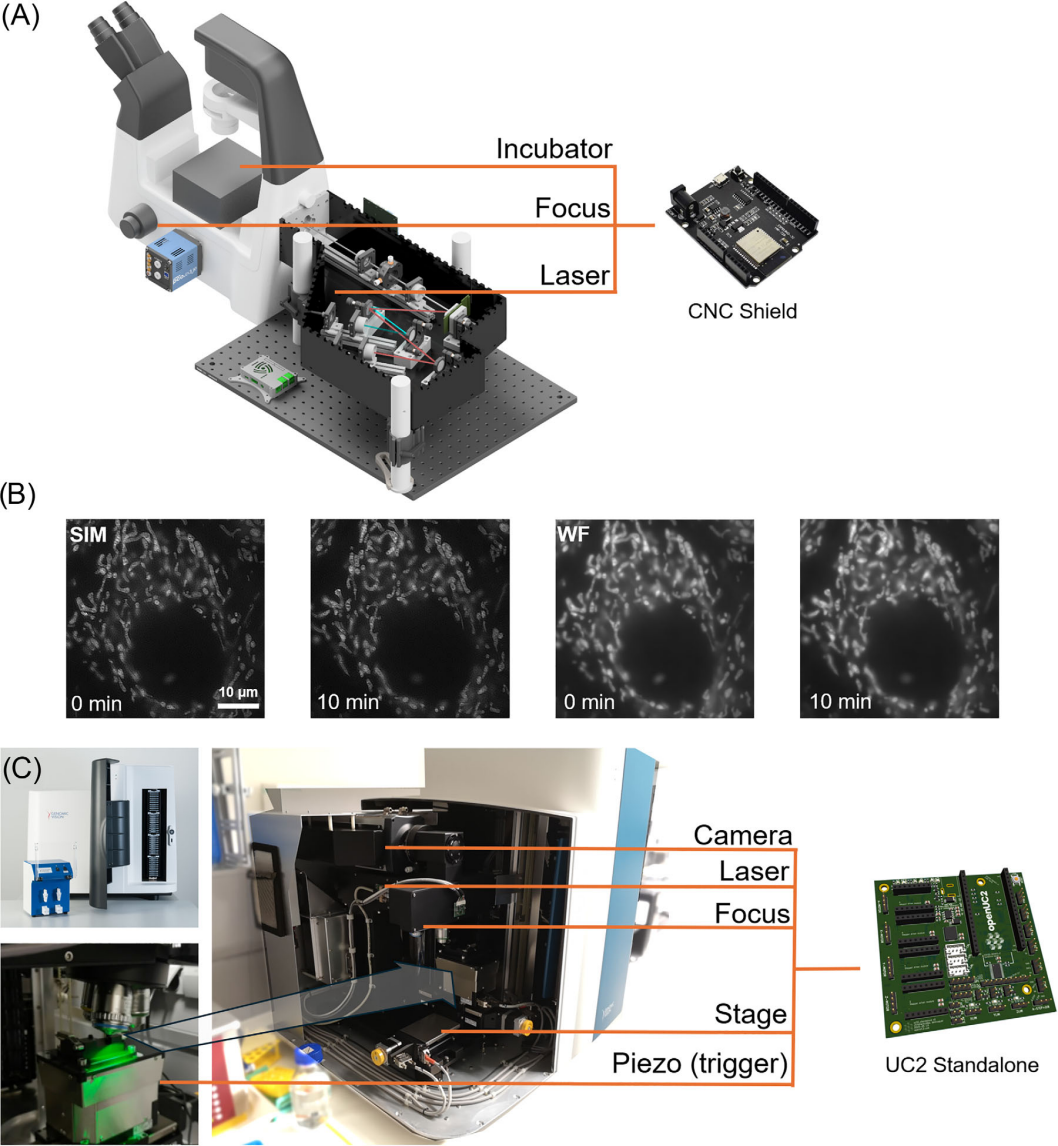
Use cases with customized microscopes. (A) An open‐source structured illumination microscopy extension controlled by the firmware, (B) time‐lapse imaging of MitoTracker‐labelled HeLa cells acquired with the openSIMMO setup under UC2‐ESP control. SIM reconstruction (left) shows improved optical sectioning relative to the pseudo‐widefield (WF) reference (right) obtained by averaging the raw SIM frames,^20^ (C) a revived multicolour fluorescence microscope for automated slide scanning.

Wang et al.^20^, ^21^ presented an open‑source structured‑illumination extension depicted in Figure 5A driven entirely by an ESP32 Wemos D1 board in combination with the CNC Shield v3 board running UC2‑ESP ***[env:UC2_WEMOS]***. Two fibre‐coupled diode lasers (488 and 638 nm) receive TTL modulation from the board, whereas an NEMA‑17 stepper motor that was coupled to the microscope's fine‑focus knob translates the objective for axial focus stacking. A repurposed 3D‑printer heater bed keeps the sample chamber at 37°C, its temperature held by an on‑board PID loop with a temperature sensor. The same firmware was fully integrated into the automated SIM imaging workflow, where a dedicated controller was responsible to trigger time‑lapse sequences and 3D stacks.^22^ To illustrate the time‐lapse capabilities of this setup, we acquired live‐cell imaging of HeLa cells stained with MitoTracker Green (Figure 5B). The UC2‐ESP firmware coordinated laser TTL gating and camera triggering throughout the time series. After SIM reconstruction, the optical resolution as well as sectioning improvement of structured illumination is apparent when compared to the pseudo‐widefield reference obtained by averaging the raw SIM frames. Key actuators/sensors: 2 × TTL‑gated lasers, 1 × *Z*‑axis stepper, 1 × thermistor/heater pair. PIO Firmware Config: ***[env:UC2_WEMOS]***.

### 4.2 Reviving scrap‐heap microscopes

The Genomic Vision (FiberVision) microscope (Figure 5C) became unusable after vendor software support was discontinued, despite its intact mechanical and optical subsystems. UC2‐ESP was used to regain control of the motorized *XYZ* stage (Physik Instrumente) through standard STEP/DIR stepper interfaces and a customized adapter cable to bridge the UC2 ESP32 board with their stepper motors. The original fluorescence light engine (Lumencor, Spectra X 6s) was re‐integrated via its RS‐232 protocol bridged by the ESP32 UART, whereas additional extensions, such as objective turret drivers and filter revolvers (SmarAct), were added over their proprietary USB interface. Once linked to ImSwitch, the revived instrument could perform automated routines, including multi‐plane *Z*‐stacks and tiled acquisitions. This case demonstrates how UC2‐ESP enables the sustainable repurposing of discontinued commercial systems into flexible, Python‐controlled imaging platforms, thereby extending hardware lifetimes and reducing e‐waste. PIO Firmware Config: ***[env:UC2_3]***.

### 4.3 Cost‑effective single‑molecule localization and modular light‑sheet microscopy

**FIGURE 6:**
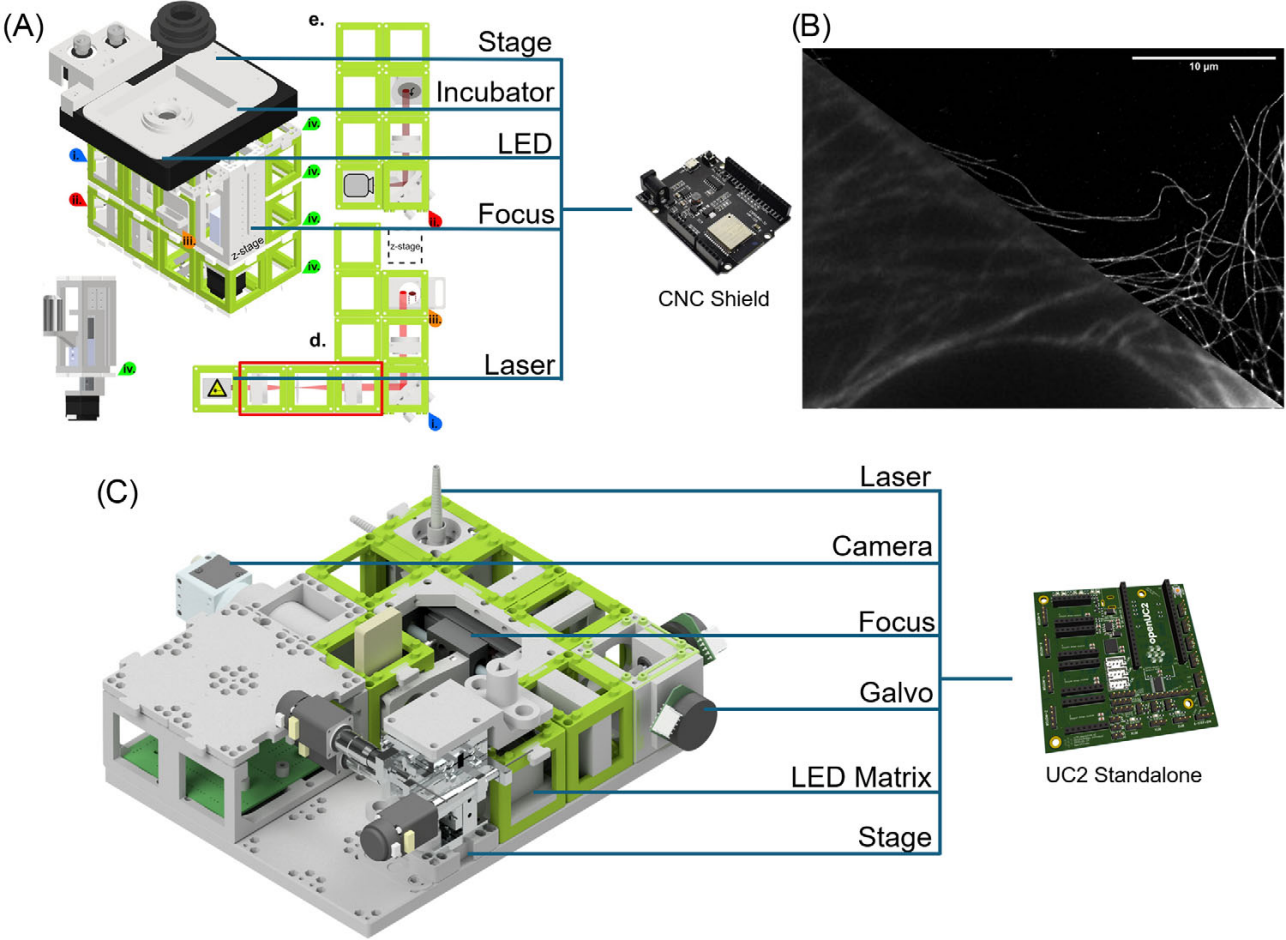
Use cases with UC2 cube‐based fluorescence microscopy systems: (A) dSTORM microscope, (B) comparison image between widefield and dSTORM reconstruction acquired with the single‐molecule localization setup under UC2‐ESP control,^23^ (C) light‐sheet microscope.

Zehrer et al. combined high‑numerical‑aperture objectives with low‑cost lasers and opto‐mechanics to create a single‐colour super‑resolution system using STORM (stochastic optical reconstruction microscopy) capable of single‑particle tracking (Figure 6A). A low‐cost high‑power laser diode and a white LED are switched consecutively via ESP‑generated TTL signals to acquire dSTORM and brightfield illumination images; three TMC‑2209 stepper channels drive the *XY* translation stage and *Z* focus. This is again orchestrated in ImSwitch,^11^ a Python interface that grants full access to the functions of the board. Alternatively, the same setup can be controlled from Micro‐Manager with the corresponding device driver. To demonstrate the super‐resolution capability of the UC2‐ESP controlled single‐molecule localization setup, Figure 6B shows a representative dSTORM reconstruction of Alexa Fluor 647‐labelled microtubules in fixed CV‐1 cells. The reconstruction was generated from 30,000 frames acquired under UC2‐ESP‐coordinated laser TTL switching and *XY* stage positioning. The reconstruction resolves individual microtubule filaments that appear not clearly resolved in the widefield image, confirming that the firmware reliably sustains the prolonged acquisition sequences required for single‐molecule localization microscopy. Key actuators/sensors: 1 × TTL‑laser, 1 × white‑LED, 3 × steppers with end‑stops, PIO Firmware Config: ***[env:UC2_WEMOS]***.

Multiple labs pair UC2‑ESP with ImSwitch for customized light‑sheet setups constructed from UC2 cubes as in Figure 6B. A motorized *XYZ* stage carries the specimen through a static laser sheet whose intensity is TTL‑modulated; another stepper adjusts detection focus. A ring of NeoPixels provides bright‑field illumination for coarse alignment. The acquisition of the *Z*‐stack is carried out such that the beginning and the end of the motion are synchronized with the frame number so that individual frames are mapped along the stack equidistantly, allowing the software to map every camera frame to an exact *Z* coordinate. Joystick control handled by the built‐in Bluetooth stack lets users quickly centre the region of interest before handing control back to an automated Python routine. The on‐board DAC can provide fast oscillating scanning signals for galvo mirrors to generate a scanning‐based light sheet. Key actuators/sensors: 1 × TTL‑laser, 1 × LED, 4 × steppers, PIO Firmware Config: ***[env:UC2_3]***.

### 4.4 Rapid prototyping for start‑ups and educational kits

**FIGURE 7:**
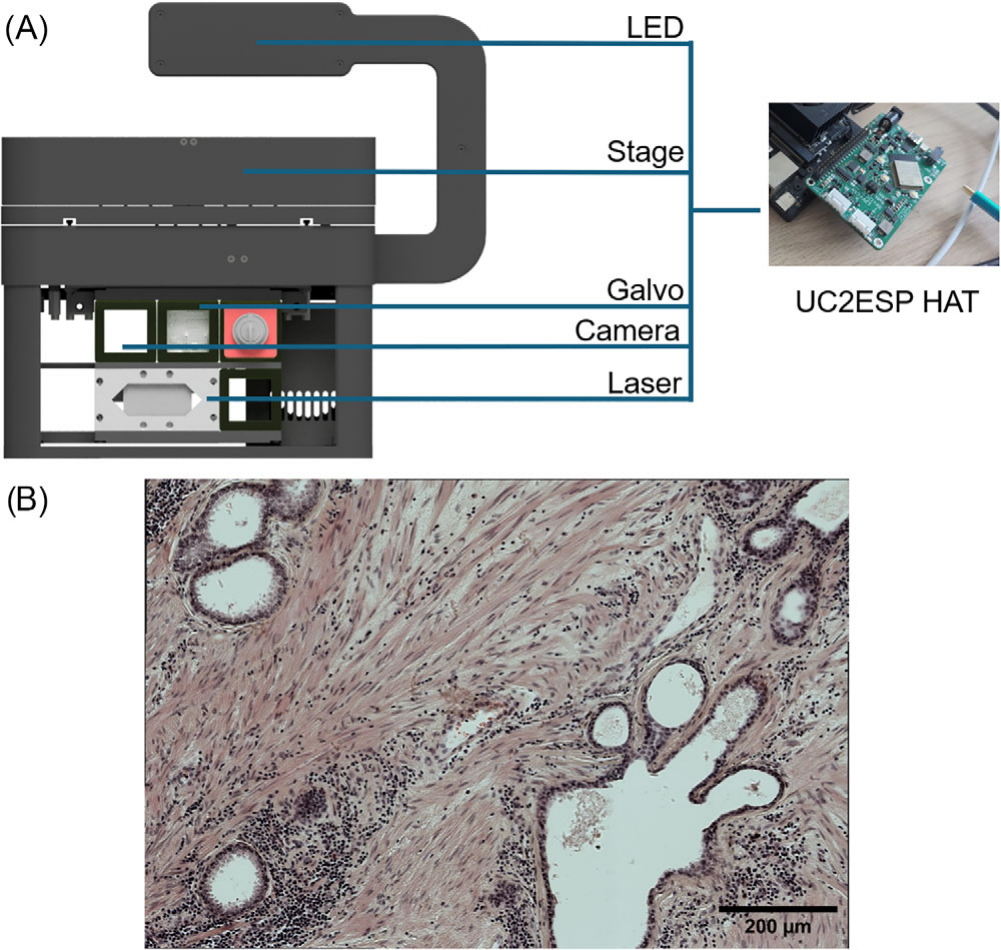
An automated microscope relies on the UC2‐ESP firmware: (A) UC2‐ESP HAT is employed as master, and each hardware has its own slave ESP for task communication; (B) a 3 × 3 tile scan of a human prostate cancer tissue section is acquired with the automated microscope under UC2‐ESP control.

Several early‑stage companies adopted UC2‑ESP to shorten their prototyping cycle. openUC2 contributed with a firmware update that extended the CAN implementation with the ISO‑TP‑enabled CAN interface, so a single twisted pair now carries commands to satellite boards controlling stage‑top incubators, motors or NeoPixel illumination rings (Figure 7). Because every firmware flavour shares the same JSON/REST API, hardware variants can be swapped without changing higher‑level code or GUIs. Seeed Studio likewise ships an educational microscope that leverages the same core to drive auxiliary pumps and RGB arrays for classroom demonstrations. Off‑loading closed‑loop temperature or pump control to tiny ESP32S3 (Xiao) CAN satellites frees the main ESP32 for high‑bandwidth USB and Wi‑Fi tasks, reducing CPU load on the main board so that USB and Wi‐Fi communication remain responsive under sustained operation. PIO Firmware Config: ***[env:UC2_3_CAN_HAT_Master, env:seeed_xiao_esp32c3_can_slave_motor]***.

## 5 CONCLUSION

We believe this firmware fills a gap between inexpensive open‐source microscopes and the growing community focused on ‘smart’ microscopy techniques. Rather than prioritizing minimum latency, we emphasize modularity, enabling researchers to mix and match different actuators, sensors and communication interfaces. In addition to supporting professional experiments such as temperature regulation or advanced scanning routines, our system is also well‐suited for teaching and demonstration purposes, offering accessible ways to learn about automated microscopy.

Future development will focus on expanding bus‐based communication (e.g., I^2^C, CAN) to incorporate additional specialized hardware components such as spatial light modulator. By distributing tasks across multiple boards, we can further improve real‐time performance and reduce the computational burden on the main MCU. We anticipate that this flexible, open‐source approach to firmware development will continue to stimulate innovation in the broader microscopy community, allowing rapid prototyping and iterative improvement of custom experimental setups.

## CONFLICT OF INTEREST STATEMENT

B.D. is co‐founder of the company openUC2 that builds and distributes open‐source microscopes. Both B.D. and H.W. are shareholders of that company. All other authors declare no conflicts of interest.

## Supporting information



Supporting Information

## Data Availability

**Table:** 

Description	Link
Firmware Repository (UC2‐ESP)	https://github.com/youseetoo/uc2‐esp32
Online‐based Firmware Flashing	https://youseetoo.github.io/
Online‐based Firmware Testing	https://youseetoo.github.io/indexWebSerialTest.html
Python interface for UC2‐ESP Firmware (UC2‐REST)	https://github.com/openUC2/UC2‐REST
Configuration Files for different board configurations; Configuration header and platformio.ini file	e.g. https://github.com/youseetoo/uc2‐esp32/blob/2e8c58d836c9aa9ce97831c19465f411cf8b873e/main/config/UC2_3_CAN_HAT_Master/PinConfig.h And https://github.com/youseetoo/uc2‐esp32/blob/2e8c58d836c9aa9ce97831c19465f411cf8b873e/platformio.ini#L654 e.g. https://github.com/youseetoo/uc2‐esp32/blob/2e8c58d836c9aa9ce97831c19465f411cf8b873e/main/config/UC2_3_CAN_HAT_Master/PinConfig.h And https://github.com/youseetoo/uc2‐esp32/blob/2e8c58d836c9aa9ce97831c19465f411cf8b873e/platformio.ini#L654
