# Supplementary material for: UC2‐ESP: A general‐purpose framework for open‐source microscopy control
Source: J Microsc. 2026 Jul 21;303(3):249–62. doi: 10.1111/jmi.70147 (PMC13535877; doi:10.1111/jmi.70147)
Supplement: Supplementary file 1 [file JMI-303-249-s001.docx]

## **Supplementary**

### Legend — UC2-ESP Bus Layers

- **GPIO** = native MCU pins (STEP/DIR, PWM, digital I/O)
- **I²C / SPI** = onboard or remote peripheral link
- **CAN** = remote module over CAN-FD network
- **Analog** = voltage or current output from DAC

### Table of available components

| Component | Category | Key operations / functions | Representative commands | Interface |
| --- | --- | --- | --- | --- |
| Stepper motor (X / Y / Z / A) | Actuator | relative / absolute move  continuous isforever run  stop, enable/disable driver  setpos, softlimits, dual-axis sync  high-level stagescan sweeps | /motor_act,  motor_get  /home_act (homing) | Native STEP/DIR GPIO  I²C (remote driver)  CAN (remote module) |
| Linear-encoder | Sensor | read position  PID assisted moveP for feedback-looped positioning  calibration calibration of step/distance relation, setup | /linearencoder_get, /linearencoder_act | A/B, I²C |
| Incremental encoder | Sensor | read position  zero / calibrate  use calliper-based absolute positioning | /encoder_get  /encoder_act | GPIO |
| Home / end-stop | Sensor | initiate homing move  set polarity, timeout | /home_act  /home_get | GPIO  (motor/digitalin) |
| Laser driver (0-3) | Actuator | analogue power LASERval  anti-speckle modulation (pwm variation, amplitude/period)  PWM/servo freq set | /laser_act  /laser_get | PWM GPIO / DAC |
| LED array (WS2812 ring / matrix / strip) | Actuator | individual RGB, rings, halves, circles, off  global intensity | /ledarr_act  /ledarr_get | GPIO (1-wire) |
| Digital output (trigger) | Actuator | assign pin  edge-timed trigger (DelayOn/Off)  reset trigger | /digitalout_set, /digitalout_act | GPIO |
| Digital input | Sensor | read pin state | /digitalin_get | GPIO |
| DAC channel | Actuator | set fixed value  waveform (frequency,offset,amplitude, shape: sine, rectangle, triangle) | /dac_act  /dac_act_fct | I²C / SPI |
| Galvo scanner | Actuator | raster scan (min/max, step, dwell time, frames) | /galvo_act  /galvo_get | Analog ±10 V via DAC |
| Heating control | Actuator + Sensor | PID on/off, set Kp/Ki/Kd, target °C  read temperature  ds18b20 temperature sensor | /heat_act  /heat_get | GPIO (PWM) + lasercontroller |
| Objective changer | Actuator | calibrate (dir, polarity)  toggle, explicit move  set slots x1/x2, focus z1/z2 | /objective_act, /objective_get | Stepper GPIO  (motor+digitalin) |
| Rotator stage | Actuator | multi-axis rotate moves  use 28byj-48 motors | /rotator_act  /rotator_get | Stepper GPIO |
| TMC driver (per axis) | Actuator config | micro-step, RMS current  stall-guard, cool-step params  control via UART | /tmc_act  /tmc_get | UART |
| CAN-bus node | Comm. | set / query node address  restart, motor proxy cmds  handle communication via ISO-TP | /can_act  /can_get | CAN (TWAI) |
| I²C-bus scan | Comm. | enumerate attached devices  send messages  read messages | /i2c_get | I²C |
| Parallel bus | Comm. | data transfer |  |  |
| System state | Sensor | heap, busy, debug, restart, delay  get build information | /state_get  /state_act | — |
| Focus-scan routine (motor + LED + trigger) | Composite scan | axial sweep Z-start → nZ steps  timed pre/trigger/post delays  LED illumination mask | /motor_act -> focusscan | Stepper GPIO + One-wire GPIO |
| Stage-scan routine (motor + laser + LED + trigger) | Composite scan | raster XY grid  per-pixel trigger, LED or laser patterns  high-speed coordinated motion | /motor_act -> stagescan | Stepper GPIO + Analog (DAC) + One-wire GPIO |

### Data availability

| Description | Link |
| --- | --- |
| Firmware Repository (UC2-ESP) | https://github.com/youseetoo/uc2-esp32 |
| Online-based Firmware Flashing | https://youseetoo.github.io/ |
| Online-based Firmware Testing | https://youseetoo.github.io/indexWebSerialTest.html |
| Python interface for UC2-ESP Firmware (UC2-REST) | https://github.com/openUC2/UC2-REST |
| Configuration Files for different board configurations; Configuration header and platformio.ini file | e.g. <https://github.com/youseetoo/uc2-esp32/blob/2e8c58d836c9aa9ce97831c19465f411cf8b873e/main/config/UC2_3_CAN_HAT_Master/PinConfig.h>  And <https://github.com/youseetoo/uc2-esp32/blob/2e8c58d836c9aa9ce97831c19465f411cf8b873e/platformio.ini#L654> |

### Competing Interests

B.D. is co-founder of the company openUC2 that builds and distributes open-source microscopes. Both B.D. and H.W. are shareholders of that company. All other authors declare no competing interests.
